# Gut Microbiome-Mediated Neuroprotective Effect of the Mediterranean Diet: A Narrative Review of Cognitive Aging, Alzheimer’s Disease, and Parkinson’s Disease

**DOI:** 10.3390/nu18193146

**Published:** 2026-09-24

**Authors:** Kundun Kamalakar, Christa Akerele, Ezequiel Lafont, Lauren R. Koralnik, Caitlin Gilman, Jose Clemente Litran, Jakleen Lee, Jessica Robinson-Papp, Dolores Malaspina

**Affiliations:** 1 Departments of Psychiatry and Pharmacology, Institute of Translational Medicine, Icahn School of Medicine at Mount Sinai, New York City, NY 10029, USA; kundun.kamalakar@mssm.edu (K.K.);; 2 Vagelos College of Physicians and Surgeons, Columbia University, New York City, NY 10032, USA; 3 Albert Einstein College of Medicine, Bronx, NY 10461, USA; 4 Department of Pediatrics, Albert Einstein College of Medicine, New York City, NY 10461, USA; 5 Departments of Genetics and Genomic Sciences and Immunology, Icahn School of Medicine at Mount Sinai, New York City, NY 10029, USA; 6 Icahn School of Medicine at Mount Sinai, New York City, NY 10029, USA; 7 Department of Neurology, Icahn School of Medicine at Mount Sinai, New York City, NY 10029, USA

**Keywords:** cognitive aging, dementia, Alzheimer’s disease, Parkinson’s disease, neuroprotection, neurodegeneration, Mediterranean diet, gut–brain axis, microbiome

## Abstract

**Background**: Alzheimer’s disease (AD) and Parkinson’s disease (PD) are major neurodegenerative disorders for which disease-modifying therapies are limited. Increasingly, evidence suggests that diet influences neurodegenerative risk through interactions among the gut microbiome, immune system, and brain. The Mediterranean diet (MD) is associated with improved cognitive health, but the mechanisms underlying its potential neuroprotective effects remain incompletely understood. **Objective:** In this narrative review, evidence on microbiome-mediated mechanisms linking MD adherence to neuroprotection was synthesized, and how Mediterranean dietary components influence microbial ecology, metabolites, immune regulation, and neuroinflammation relevant to cognitive aging, AD, and PD was evaluated. **Methods:** Evidence from experimental, observational, and clinical studies was reviewed, focusing on microbial metabolites, intestinal barrier integrity, immune regulation, and inflammatory pathways. **Discussion**: Mediterranean dietary components, including fiber, polyphenols, whole grains, legumes, nuts, extra-virgin olive oil, and omega-3 fatty acids, may promote beneficial microbial communities and metabolite production. Short-chain fatty acids, secondary bile acids, indole derivatives, and phenolic metabolites may strengthen the intestinal and blood–brain barriers, regulate immune responses, and suppress neuroinflammation. Conversely, dysbiosis may increase intestinal permeability, lipopolysaccharide translocation, systemic inflammation, and microglial activation. However, evidence remains heterogeneous and largely observational or experimental. **Conclusions:** The MD may promote neuroprotection through interconnected diet–microbiome–immune pathways. Longitudinal and intervention studies integrating microbiome, metabolomic, inflammatory, neuroimaging, and cognitive measures are needed to establish causality and advance precision nutrition strategies to prevent neurodegenerative disease.

## 1. Introduction

Neurodegenerative diseases, including Alzheimer’s disease (AD) and Parkinson’s disease (PD), are major global public health challenges because of their increasing prevalence, effects on quality of life, and substantial societal burden. Both disorders are strongly associated with advancing age and with population aging is expected to increase the number of individuals affected by neurodegenerative disease worldwide is expected to increase [1,2,3]. The growing burden of neurological and neurodegenerative disorders highlights the need for effective strategies to preserve brain health and reduce the risk of disease across the entire lifespan [1,3].

Beyond their effects on individuals and families, neurodegenerative diseases impose substantial economic and healthcare burdens. As AD and PD progress, costs increase due to medical visits, diagnostic testing, medications, hospitalizations, long-term supportive care, and institutional services [3,4]. These conditions also generate indirect costs through reduced workforce participation, lost productivity, and unpaid caregiving by family members. The expanding clinical and economic burdens of neurodegenerative disease therefore underscore the need for preventive strategies that can delay disease onset or slow progression [3,4].

The absence of curative therapies for AD and PD positions prevention as a critical strategy for reducing future disease burden. Preventive approaches increasingly focus on modifiable risk factors associated with cognitive decline and neurodegeneration, including hypertension, diabetes, obesity, smoking, physical inactivity, and other lifestyle-related factors [5,6]. Evidence indicates that addressing modifiable risk factors by engaging in regular physical activity, improving dietary patterns, and effectively managing chronic conditions may reduce the risk or delay the onset of dementia by influencing biological processes involved in neurodegeneration before substantial and irreversible neuronal damage occurs [6]. Identifying accessible, evidence-based lifestyle interventions is therefore a priority for promoting healthy brain aging and reducing the growing burden of neurodegenerative disease.

Diet is one of the most promising modifiable factors for supporting neurological health and reducing neurodegenerative risk. Among dietary interventions, the Mediterranean diet has consistently demonstrated neuroprotective associations and has become a cornerstone of lifestyle-based approaches for cognitive health [7]. Characterized by a predominantly plant-based dietary pattern, the Mediterranean diet emphasizes high consumption of fruits, vegetables, whole grains, legumes, nuts, and seeds, with extra-virgin olive oil serving as the primary source of dietary fat [8]. Moderate intake of fish, poultry, and fermented dairy products is encouraged, while red and processed meats are limited. This dietary pattern provides abundant bioactive compounds, including omega-3 fatty acids, polyphenols, vitamins, and minerals, which collectively support brain health by reducing oxidative stress, regulating inflammation, improving vascular function, and preserving neuronal integrity [8].

A growing body of epidemiological and clinical evidence indicates that sustained adherence to the Mediterranean diet is associated with slower cognitive decline, improved memory and executive function, and reduced risk of Alzheimer’s disease and dementia [9]. Proposed mechanisms underlying these neuroprotective effects include reduced neuroinflammation, enhanced antioxidant capacity, improved cerebral blood flow, and preservation of synaptic function [8,9]. Importantly, emerging evidence suggests that these benefits may extend to individuals with increased genetic susceptibility to AD, including carriers of the APOE-ε4 allele, who demonstrate slower cognitive decline with greater adherence to Mediterranean dietary patterns [7,10]. Collectively, these findings highlight the Mediterranean diet as an accessible, evidence-based strategy for promoting healthy cognitive aging.

The gut microbiome is a complex ecosystem composed of trillions of microorganisms residing throughout the gastrointestinal tract that play a critical role in maintaining intestinal, immune, and neurological homeostasis. Communication between the gut microbiome and the central nervous system occurs through the gut–brain axis, a bidirectional signaling network involving microbial metabolites, immune mediators, endocrine pathways, and neural communication through the vagus nerve [11,12]. Through these pathways, intestinal microorganisms produce bioactive compounds, including neurotransmitter-related molecules, serotonin, dopamine, gamma-aminobutyric acid (GABA), and short-chain fatty acids (SCFAs), which influence neuronal function, cognition, mood regulation, and immune activity [11]. Furthermore, because the gastrointestinal tract contains a substantial proportion of the body’s immune cells, alterations in the intestinal environment can significantly influence systemic inflammatory responses and downstream neurological processes [12].

Disruption of the gut microbiome, commonly referred to as dysbiosis, has emerged as a potential contributor to the development and progression of neurodegenerative diseases, including AD and PD [13,14,15] [Table 1]. Dysbiosis is characterized by alterations in microbial composition, reduced microbial diversity, and impaired microbial function, which can compromise intestinal barrier integrity and increase intestinal permeability [14]. This disruption may allow bacterium-derived products, including lipopolysaccharides (LPSs) and other inflammatory molecules, to enter the systemic circulation and promote chronic low-grade inflammation [14]. Systemic inflammation can subsequently influence the central nervous system by activating microglia, increasing oxidative stress, disrupting blood–brain barrier function, and promoting neuronal injury, all of which are implicated in the pathogenesis of neurodegenerative diseases [16].

Diet represents one of the strongest environmental determinants of gut microbial composition and function [16,17]. Dietary patterns characterized by high consumption of refined sugars, saturated fats, and ultra-processed foods have been associated with reduced microbial diversity, increased intestinal permeability, and inflammatory signaling. In contrast, the Mediterranean diet provides abundant dietary fiber, polyphenols, omega-3 fatty acids, and other bioactive compounds that promote beneficial microbial communities and enhance the production of neuroprotective metabolites, including SCFAs [16] [Table 1]. These microbiome-derived metabolites contribute to intestinal barrier integrity, regulate immune function, reduce systemic inflammation, and may influence neuroinflammatory pathways involved in cognitive decline and neurodegeneration.

Although previous reviews have been conducted to examine the relationship between diet, cognition, and the gut microbiome, important gaps remain in the current literature. Existing reviews have largely focused on associations between dietary patterns and cognitive outcomes, summarized the neuroprotective effects of the Mediterranean diet, or independently examined the role of gut microbiota in neurological disease. While these studies have advanced our understanding of each component, few have integrated these fields by critically evaluating how Mediterranean diet-induced alterations in gut microbial composition and function contribute to shared biological mechanisms underlying neurodegeneration. Furthermore, few reviews have directly compared microbiome-mediated pathways across both Alzheimer’s disease and Parkinson’s disease, despite increasing evidence that microbial dysbiosis, intestinal barrier dysfunction, immune dysregulation, and chronic neuroinflammation are overlapping mechanisms in both disorders.

Addressing this knowledge gap is essential for developing a more comprehensive understanding of the gut–brain axis and identifying potential dietary and microbiome-based interventions for preventing neurodegenerative disease. A unified framework integrating dietary patterns, microbial ecology, immune regulation, and neuroinflammatory pathways may provide new insights into strategies capable of delaying disease onset or slowing progression.

This narrative review synthesizes current evidence on the microbiome-mediated mechanisms underlying the neuroprotective effects associated with adherence to the Mediterranean diet. How Mediterranean dietary patterns influence gut microbial ecology, microbial metabolites, immune regulation, and neuroinflammatory signaling, thereby affecting cognitive aging and the development and progression of Alzheimer’s and Parkinson’s disease, is also critically evaluated. Further, inconsistencies in the literature are identified, the strength of mechanistic evidence is evaluated, and future research priorities for precision nutrition and microbiome-based therapeutic strategies are proposed.

### Literature Search Methodology

A narrative literature search was conducted to identify experimental, observational, and clinical evidence relevant to the relationship between the Mediterranean diet, gut microbiome, microbiota–gut–brain axis, inflammation, and neurodegenerative disease, with particular emphasis on Alzheimer’s disease (AD) and Parkinson’s disease (PD). Searches were conducted in PubMed/MEDLINE, Scopus, and Web of Science from 2012 through 2026 for current research references. We also used some historical papers as old as 1990 to establish chronological progression. Principal search terms included combinations of keywords and controlled vocabulary related to the “Mediterranean diet,” “Mediterranean dietary pattern,” “gut microbiome,” “gut microbiota,” “microbiota–gut–brain axis,” “microbial metabolites,” “short-chain fatty acids,” “intestinal barrier,” “blood–brain barrier,” “neuroinflammation,” “microglia,” “Alzheimer’s disease,” and “Parkinson’s disease.” Experimental and mechanistic studies, observational studies, clinical studies, and relevant reviews were prioritized based on their relevance to the proposed biological pathways and human disease outcomes. Reference lists of key publications were also examined to identify additional relevant studies, and citation searching was used where appropriate to identify related and subsequently published evidence. Because this was a narrative review, studies were selected based on relevance to the review’s mechanistic framework rather than according to formal systematic-review eligibility criteria.

## 2. Mediterranean Diet and Gut Microbial Ecology

### 2.1. Changes in Gut Microbiota

#### 2.1.1. The Healthy Gut Microbiome

A healthy gut microbiome is a diverse, resilient, and metabolically active ecosystem composed of trillions of microorganisms that contribute to nutrient metabolism, immune regulation, and host physiology. Rather than having a fixed microbial composition, the microbiome continuously adapts to environmental influences, including diet, pathogen exposure, and antibiotic use, while maintaining functional interactions with the host [18,19].

Although microbial composition varies substantially among individuals, different microbial taxa can perform overlapping metabolic functions, a property known as functional redundancy that contributes to ecosystem stability and resilience [2,19]. A key function of the gut microbiome is the fermentation of nondigestible dietary carbohydrates into SCFAs, including acetate, propionate, and butyrate. These metabolites support intestinal epithelial metabolism, regulate immune responses, and contribute to communication along the microbiota–gut–brain axis [20].

A balanced microbiome also supports intestinal barrier integrity and immune homeostasis by promoting mucus production, maintaining epithelial tight-junction function, and limiting the translocation of pathogens and pro-inflammatory microbial products into the circulation [18]. Through continuous interactions with intestinal immune cells, the microbiome helps maintain immune tolerance while preventing excessive inflammatory activation. Collectively, these functions support intestinal and systemic homeostasis and provide a biological foundation through which gut microbial health may influence cognitive aging and the risk of neurodegenerative disease [18].

#### 2.1.2. Mediterranean Diet as a Modulator of Microbial Ecology

The Mediterranean diet can influence gut microbial ecology by promoting microbial diversity and favoring bacterial taxa associated with intestinal and systemic health [21,22,23]. Its high intake of whole grains, legumes, fruits, vegetables, nuts, and extra-virgin olive oil provides fermentable carbohydrates, fiber, and polyphenols that can shape microbial composition and metabolic activity [21,22]. These diet-associated changes may support a more diverse and metabolically resilient microbial ecosystem that influences host metabolic and immune function.

Among the taxa associated with Mediterranean-style dietary patterns are *Bifidobacterium*, *Lactobacillus*, *Faecalibacterium prausnitzii*, and *Akkermansia muciniphila* [21]. Several of these microorganisms utilize dietary carbohydrates or fermentable substrates and contribute to the production of metabolites, including SCFAs, that support intestinal homeostasis. In particular, *F. prausnitzii* is an important butyrate-producing bacterium, whereas *A. muciniphila* has been associated with intestinal barrier integrity and metabolic health [21,22]. Although the abundance of individual taxa varies across studies and populations, adherence to the Mediterranean diet is generally associated with microbial profiles characterized by greater diversity and enrichment of bacteria linked to favorable metabolic and inflammatory outcomes.

Conversely, dietary patterns characterized by lower intake of plant-based foods and higher consumption of processed foods and animal products have been associated with microbial features linked to inflammation and dysbiosis. In longitudinal population data, healthier dietary patterns were associated with a greater abundance of microbial taxa and functions considered anti-inflammatory, whereas less favorable dietary patterns were associated with pro-inflammatory microbial features [24]. Thus, rather than uniformly increasing or decreasing individual bacterial species, the Mediterranean diet appears to influence the overall composition and functional capacity of the gut microbiome [24]. These ecological changes provide a potential foundation for the microbiome-mediated metabolic, immune, and neuroprotective mechanisms discussed in subsequent sections of this review.

#### 2.1.3. Functional Beneficial Outcomes of Microbiome Remodeling

Mediterranean diet-associated remodeling of the gut microbiome can produce functional changes that support intestinal and systemic homeostasis. A key outcome is increased microbial fermentation of dietary fiber, generating SCFAs, particularly acetate, propionate, and butyrate [25,26]. These metabolites mediate host–microbiome communication and influence metabolic, immune, and intestinal processes.

Among SCFAs, butyrate is particularly important for intestinal barrier function. As a major energy substrate for colonocytes, it supports epithelial homeostasis, tight-junction integrity, and mucosal defense [26]. SCFAs may therefore reduce intestinal permeability and limit translocation of microbial products such as LPS into the circulation, reducing inflammatory exposure [25,26].

SCFAs also regulate immune responses through G-protein-coupled receptors and histone deacetylase inhibition, including pathways affecting regulatory T-cell activity and inflammatory signaling [25,26]. Collectively, increased SCFA production, improved epithelial integrity, and immune regulation provide a biological basis for the potential effects of Mediterranean diet-associated microbiome remodeling on systemic and brain health. These mechanisms are examined in greater detail in the following sections.

### 2.2. Mediterranean Dietary Components

The Mediterranean diet provides fermentable fiber, polyphenols, unsaturated fatty acids, and fermented foods that shape gut microbial composition and metabolic activity. Many of these compounds reach the colon, where they are metabolized by gut microorganisms into bioactive metabolites that influence intestinal barrier integrity, immune signaling, and systemic inflammation. These diet–microbiome interactions provide a potential mechanistic link between Mediterranean diet adherence and neuroprotection. Building upon these principles, the Mediterranean-DASH Intervention for Neurodegenerative Delay (MIND) diet incorporates additional emphasis on foods associated with cognitive health, including leafy green vegetables, berries, and nuts, and reduced intake of processed foods while preserving many Mediterranean dietary components [27]. Emerging evidence suggests that adherence to MIND diet patterns may further support neuroprotection through overlapping mechanisms involving reduced oxidative stress, inflammation, and metabolic dysfunction [27].

#### 2.2.1. Dietary Fiber

Plant-based foods, including whole grains, legumes, nuts, seeds, fruits, and vegetables, are major sources of dietary fiber and form the foundation of the Mediterranean diet. Because fiber resists digestion in the small intestine, it reaches the colon, where gut microorganisms ferment it into SCFAs, including acetate, propionate, and butyrate [28]. SCFAs support colonocyte metabolism, intestinal barrier integrity, and immune regulation while providing substrates that favor fiber-fermenting microbial communities [28,29]. Consequently, higher fiber intake is generally associated with greater microbial diversity and enrichment of bacteria capable of producing beneficial metabolites [28]. Through these microbiome-mediated effects, dietary fiber may reduce intestinal and systemic inflammation and support gut–brain homeostasis.

#### 2.2.2. Polyphenols

Polyphenols are plant-derived bioactive compounds abundant in berries, grapes, tea, cocoa, and extra-virgin olive oil [30]. Many escape digestion in the small intestine and reach the colon, where gut microorganisms convert them into smaller phenolic metabolites that may have greater bioavailability and biological activity [30,31]. These metabolites can influence oxidative stress, inflammatory signaling, and endothelial function, potentially contributing to neuroprotection [32].

The relationship between polyphenols and the gut microbiome is bidirectional. Polyphenols may exert prebiotic-like effects by promoting beneficial taxa such as *Lactobacillus* and *Bifidobacterium* while suppressing some pro-inflammatory microorganisms [33]. Conversely, microbial composition determines how efficiently individual polyphenols are metabolized and which bioactive compounds are produced [30,33]. Thus, polyphenol-rich Mediterranean foods may influence gut microbial ecology, while microbial metabolism determines the downstream biological effects of these compounds.

#### 2.2.3. Extra-Virgin Olive Oil

Extra-virgin olive oil (EVOO) is a hallmark of the Mediterranean diet and provides both monounsaturated fatty acids (MUFAs) and bioactive phenolic compounds. Its predominant fatty acid, oleic acid, contributes to metabolic and intestinal health, while phenolics such as hydroxytyrosol and oleuropein exhibit antioxidant and anti-inflammatory properties [34,35].

Importantly, EVOO may influence the gut microbiome through its phenolic compounds, which can exert prebiotic-like effects and promote beneficial microbial taxa [36]. These microbial changes may increase SCFA production and support epithelial barrier function and immune homeostasis. EVOO polyphenols may also directly influence intestinal epithelial and inflammatory pathways, potentially reducing intestinal permeability and systemic exposure to microbial endotoxins [36]. Thus, EVOO may contribute to Mediterranean diet-associated benefits through complementary effects on microbial composition, microbial metabolites, and intestinal barrier integrity.

#### 2.2.4. Omega-3 Fatty Acids and Fish

Fatty fish, including salmon, sardines, mackerel, and anchovies, are sources of long-chain omega-3 polyunsaturated fatty acids (PUFAs), eicosapentaenoic acid (EPA), and docosahexaenoic acid (DHA) [37]. EPA and DHA alter inflammatory lipid signaling and serve as precursors to specialized pro-resolving mediators, including resolvins, protectins, and maresins, which promote resolution of inflammation and immune homeostasis [38,39].

Omega-3 fatty acids may also influence gut microbial composition and function. Experimental and human studies indicate that omega-3 intake can modify microbial communities and metabolites associated with intestinal barrier integrity and inflammatory signaling, although effects vary according to baseline diet, microbiome composition, dosage, and metabolic status [40,41,42]. Thus, omega-3 fatty acids may contribute to Mediterranean diet-associated anti-inflammatory effects through both lipid-mediated pathways and modulation of the gut microbiome, although further human studies are needed to establish their specific contribution to gut–brain signaling.

#### 2.2.5. Whole Grains

Whole grains, including oats, barley, brown rice, and whole wheat, provide fermentable carbohydrates with prebiotic properties. Their fiber, β-glucans, resistant starch, arabinoxylans, and cellulose reach the colon and serve as substrates for microbial fermentation [43]. These substrates can promote beneficial bacteria and increase microbial diversity, supporting a more resilient gut ecosystem [44].

Microbial fermentation of whole-grain carbohydrates increases SCFA production, particularly butyrate, which provides energy for colonocytes and supports intestinal barrier integrity [45]. Thus, whole grains influence gut health primarily by providing substrates that shape microbial communities and increase production of metabolites involved in epithelial and immune regulation.

#### 2.2.6. Nuts

Walnuts, almonds, and pistachios provide dietary fiber, unsaturated fatty acids, and polyphenols that can interact with the gut microbiome. Portions of these compounds resist digestion and reach the colon, where they serve as substrates for microbial metabolism and the production of bioactive metabolites [46]. Walnuts also provide α-linolenic acid, a plant-derived omega-3 fatty acid that may have anti-inflammatory effects [47].

Nut consumption has been associated with changes in microbial diversity, bacterial taxa, and microbial metabolites, including SCFAs, although effects vary by nut type, dose, population, and baseline microbiome composition [47]. Through these interactions, nuts may promote microbial diversity, SCFA production, intestinal barrier integrity, and immune homeostasis, providing another pathway through which the Mediterranean diet may influence systemic and neurological health.

#### 2.2.7. Legumes

Legumes are rich in fermentable carbohydrates, dietary fiber, and resistant starch, making them important substrates for gut microbial fermentation [48]. Because these carbohydrates resist digestion in the small intestine, they reach the colon and support microbial growth and activity. Their fermentation produces SCFAs, including acetate, propionate, and butyrate, which provide energy for colonocytes and regulate epithelial and immune function [49,50].

These fermentation-derived metabolites provide a key mechanism through which legume consumption can influence gut microbial function and host physiology: legumes influence the gut microbiome by providing fermentable substrates that promote beneficial microbial activity and SCFA production. These effects may strengthen intestinal barrier function and support metabolic and immune homeostasis, linking regular legume consumption to the broader microbiome-mediated benefits of the Mediterranean diet [50].

#### 2.2.8. Fermented Dairy

Fermented dairy products, including yogurt, kefir, and some aged cheeses, provide live microorganisms and fermentation-derived compounds that can interact with the gut microbiome. Yogurt is primarily fermented by *Lactobacillus bulgaricus* and *Streptococcus thermophilus*, whereas kefir contains a more diverse community of bacteria and yeasts [51,52,53]. Fermented cheeses may also contain viable microorganisms and bioactive peptides generated during fermentation [52].

These microorganisms and fermentation products may influence resident microbial communities, microbial metabolism, and intestinal immune signaling. However, effects vary substantially according to microbial strains, fermentation methods, and whether viable organisms remain in the final product; moreover, only specific strains with demonstrated clinical benefits meet the definition of probiotics [52,54,55]. Therefore, fermented dairy may contribute to gut microbiome diversity and function, but its microbiome-mediated effects are product- and strain-specific rather than uniform across all fermented dairy foods.

## 3. Microbiome-Mediated Mechanisms of Neuroprotection

### 3.1. The Microbiota–Gut–Brain Axis

The microbiota–gut–brain axis is a bidirectional communication network connecting the gastrointestinal tract and central nervous system through neural, immune, endocrine, and metabolic pathways [56,57,58]. Neural signaling occurs primarily through the vagus nerve and enteric nervous system, while immune pathways involve cytokines and other mediators that influence systemic inflammation and neuroimmune function [56,57]. Gut microbes also contribute to endocrine and metabolic signaling through neuroactive compounds and interactions with the hypothalamic–pituitary–adrenal (HPA) axis.

Microbial metabolites, including SCFAs, further contribute to gut–brain communication by influencing barrier integrity, immune signaling, and neuronal function [56,57]. However, SCFA effects are not uniformly beneficial and vary according to metabolite, concentration, receptor signaling, tissue context, and disease state. These interconnected pathways provide a framework through which diet-induced microbiome changes may influence brain health and neurodegenerative disease risk.

### 3.2. Microbial Metabolites

Microbial metabolites—including SCFAs (acetate, propionate, and butyrate), secondary bile acids, indoles, trimethylamine N-oxide (TMAO), and phenolic metabolites—mediate gut–brain communication, immune regulation, and metabolic signaling [59,60,61]. The Mediterranean diet may influence these pathways through its fiber, polyphenol, and unsaturated fatty acid content.

SCFAs are produced through microbial fermentation of nondigestible carbohydrates, including dietary fiber and resistant starch, by bacteria such as *Faecalibacterium*, *Roseburia*, and *Bifidobacterium* [59]. Whole grains, legumes, fruits, vegetables, and nuts provide substrates for their production. However, acetate, propionate, and butyrate have distinct biological effects. Butyrate serves as a major energy source for colonocytes and can support epithelial and immune function, whereas acetate and propionate participate in systemic metabolic and immune signaling [59,60]. Propionate, in particular, may exert beneficial or pro-inflammatory effects depending on concentration, receptor signaling, tissue context, and disease state [59,60].

SCFA activity is mediated partly through receptors such as FFAR2/GPR43 and FFAR3/GPR41, as well as other cellular pathways [59,60]. Thus, increased SCFA production should not be considered inherently beneficial; effects depend on the specific metabolite and host context. Within the microbiota–gut–brain axis, SCFAs may influence intestinal and BBB integrity, immune signaling, and microglial function [60].

Secondary bile acids are produced when gut bacteria transform primary bile acids, including cholic and chenodeoxycholic acids, through processes such as deconjugation and 7α-dehydroxylation. They act through receptors including farnesoid X receptor (FXR) and Takeda G-protein receptor 5 (TGR5) to regulate metabolic and immune processes [59]. Altered bile acid metabolism has been associated with neuroinflammation and neurodegenerative disease, although effects vary among individual metabolites. The Mediterranean diet may influence these pathways by altering dietary substrates and microbial activity.

Indoles are produced through microbial metabolism of dietary tryptophan and include indole and indole-3-propionic acid (IPA). Indole derivatives can activate the aryl hydrocarbon receptor (AhR), regulating mucosal immunity, epithelial integrity, and inflammatory signaling; some, including IPA, also have antioxidant properties [62] [Table 1]. Mediterranean diet foods such as legumes, nuts, and seeds provide substrates for these pathways while supporting microbial diversity.

In contrast, trimethylamine N-oxide (TMAO) is produced through microbial and host metabolism of dietary choline, betaine, and carnitine. Gut bacteria convert these nutrients to trimethylamine (TMA), which is subsequently converted to TMAO in the liver by flavin-containing monooxygenases [63]. Elevated TMAO concentrations have been associated with metabolic and vascular dysfunction and, in some studies, with inflammatory and oxidative pathways relevant to neurodegeneration. However, TMAO should not be considered universally pro-inflammatory, as its biological effects and associations with disease may vary according to metabolic context, host factors, and underlying disease states, and its causal significance remains incompletely established. Diets high in red and processed meat may provide greater amounts of TMAO-producing substrates, whereas the Mediterranean diet emphasizes plant-based foods, fiber, polyphenols, and unsaturated fats. Thus, dietary composition may influence microbial TMAO production and downstream TMAO exposure, providing a potential link between gut microbial metabolism, systemic physiology, and neurological health without implying a direct or uniformly deleterious effect of TMAO [Table 1].

Collectively, microbial metabolites represent an important intermediary between Mediterranean diet composition, microbial activity, and host physiology. The promotion of metabolites such as SCFAs, indoles, and phenolic compounds, together with potentially lower TMAO exposure, may influence intestinal integrity, immune regulation, and metabolic signaling. These effects provide a mechanistic foundation for subsequent effects on systemic inflammation, the BBB, and neuroinflammation.

### 3.3. Intestinal Barrier Integrity

The intestinal epithelial barrier is a selective interface separating the gut lumen from systemic circulation. It permits nutrient absorption while limiting the translocation of microorganisms and microbial products [64]. Tight junction proteins, including claudins, occludin, and zonula occludens-1 (ZO-1), regulates paracellular permeability, while the mucus layer, composed primarily of mucin 2 (MUC2), limits direct contact between microbes and epithelial cells [65]. Together, these structures maintain intestinal homeostasis and immune tolerance.

These barrier functions are influenced by both diet and microbial metabolism. The Mediterranean diet may support barrier integrity through both dietary components and microbiome-derived metabolites. Dietary fibers from fruits, vegetables, legumes, and whole grains are fermented to produce SCFAs, particularly butyrate, which serves as an energy source for colonocytes and supports epithelial function [66]. SCFAs can also promote mucus production, while polyphenols and omega-3 fatty acids may reduce oxidative stress and inflammatory signaling [66].

In contrast, dysbiosis may impair these protective mechanisms. Dysbiosis can disrupt these processes by reducing SCFA production and butyrate availability, weakening epithelial tight junctions and increasing intestinal permeability. This facilitates microbial translocation, including entry of LPS into the circulation [67]. Circulating LPS can activate Toll-like receptor 4 (TLR4) and downstream NF-κB signaling, increasing production of pro-inflammatory cytokines such as TNF-α, IL-1β, and IL-6 [67].

Accordingly, Mediterranean diet-associated microbiome remodeling may help preserve barrier function. By promoting microbial diversity and SCFA-producing bacteria, the Mediterranean diet may help preserve epithelial integrity and reduce LPS translocation [26]. This may limit endotoxin-driven immune activation and chronic systemic inflammation.

These peripheral effects may subsequently influence the CNS. Intestinal barrier dysfunction and the resulting inflammatory exposure may affect the CNS by compromising BBB integrity and promoting neuroinflammation. Circulating LPS and inflammatory cytokines can increase BBB permeability and activate microglia, contributing to oxidative stress and neuronal injury [68,69]. These mechanisms have been implicated in AD, where inflammation is associated with amyloid-β and tau pathology, and PD, where neuroinflammation contributes to α-synuclein-associated pathology and dopaminergic neuronal injury [69,70,71] [Table 2].

Thus, preservation of the intestinal barrier represents an upstream mechanism through which Mediterranean diet-associated microbiome remodeling may reduce systemic inflammatory exposure and influence downstream neuroinflammatory processes [16,69].

### 3.4. Microbiome-Mediated Immune Regulation and Neuroinflammation

The gut microbiome regulates systemic immunity and neurological function through the bidirectional microbiota–gut–brain axis [72,73]. Microbial metabolites, including SCFAs, secondary bile acids, and indole derivatives, influence mucosal immunity and inflammatory signaling [73,74]. Among these, SCFAs can promote anti-inflammatory pathways and regulatory T-cell (Treg) function, while bile acids and indole metabolites contribute to immune regulation [73]. These microbiome-mediated effects help maintain peripheral immune homeostasis.

Dysbiosis can instead increase intestinal permeability and facilitate translocation of microbial products such as LPS [56,74]. These signals can activate systemic immune responses and, through effects on the BBB and neuroimmune signaling, promote microglial activation and persistent neuroinflammation [56,74]. One important mechanism through which the microbiome may influence this balance is regulation of T-cell activity.

Regulatory T cells maintain immune tolerance and limit excessive inflammation through mechanisms including secretion of interleukin-10 (IL-10) and transforming growth factor-β (TGF-β) [75,76]. In the intestine, Tregs promote tolerance toward dietary antigens and commensal microorganisms. Microbial metabolites, particularly SCFAs, can influence Treg differentiation; dietary fiber supports SCFA-producing bacteria, while butyrate can enhance Treg development through epigenetic regulation of FoxP3, a transcription factor essential for Treg stability and function [75]. Increased Treg activity may therefore promote peripheral immune homeostasis and help limit excessive inflammatory signaling.

In parallel with Treg-mediated regulation, microbial and inflammatory signals can influence broader innate immune pathways. Nuclear factor kappa B (NF-κB) is a central transcriptional regulator of innate immune and inflammatory responses. LPS, pro-inflammatory cytokines, and oxidative stress can activate NF-κB, promoting transcription of inflammatory mediators [77,78,79,80]. Although necessary for host defense, sustained activation contributes to chronic inflammation. Gut barrier dysfunction can amplify this pathway when circulating LPS activates Toll-like receptor 4 (TLR4) and downstream NF-κB signaling [78,80] [Table 1].

Mediterranean diet components may counter these processes through complementary anti-inflammatory and antioxidant effects. Polyphenols, omega-3 fatty acids, and SCFAs can modulate inflammatory signaling and oxidative stress while enhancing antioxidant defenses through pathways including nuclear factor erythroid 2–related factor 2 (Nrf2) [80]. Because sustained NF-κB activation can promote microglial activation, oxidative stress, and neuronal injury, modulation of this pathway provides a plausible link between Mediterranean diet–microbiome interactions and reduced neuroinflammation [80]. More specific inflammatory pathways, including inflammasome signaling, may also contribute to these effects; however, their role should be interpreted in the context of evidence directly linking Mediterranean diet–microbiome interactions to specific inflammasome mechanisms.

Downstream of these signaling pathways, inflammatory cytokines provide important mediators of systemic and neuroinflammatory responses. IL-6, TNF-α, and IL-1β are important mediators of chronic inflammation and neuroinflammatory signaling. Altered concentrations of these cytokines have been reported in AD and PD, where persistent inflammatory signaling can contribute to microglial activation, oxidative stress, and neuronal dysfunction [81,82,83,84]. Mediterranean diet components, particularly fiber, polyphenols, and omega-3 fatty acids, may attenuate these pathways through effects on microbial metabolites, redox balance, and immune regulation [16,37,50].

IL-1β provides an additional link between innate immune activation and neurodegeneration. Persistent IL-1β signaling can contribute to microglial activation and inflammatory neuronal injury in AD [83,84]. Rather than attributing these effects to a single inflammasome pathway, evidence supports a broader model in which Mediterranean diet-derived polyphenols and fiber-derived SCFAs modulate inflammatory and oxidative signaling that influences cytokine production [8,50].

Systemic inflammatory burden can also be reflected by circulating biomarkers such as C-reactive protein (CRP)**.** CRP is a liver-derived acute-phase protein produced primarily in response to IL-6 and is widely used as a marker of systemic inflammation [85,86]. Although elevated CRP is associated with cognitive decline and neurodegenerative disease, it primarily reflects systemic inflammatory burden rather than directly mediating neurodegeneration [85]. Mediterranean diet adherence is associated with lower CRP concentrations, potentially reflecting the effects of dietary fiber, polyphenols, and unsaturated fatty acids on inflammatory signaling [8,87]. CRP may therefore serve as an indicator of the systemic inflammatory environment influenced by diet–microbiome interactions [50].

Together, these immune and inflammatory pathways provide a potential link between microbiome remodeling and neuroinflammation. Mediterranean diet-derived fiber, polyphenols, and unsaturated fatty acids can influence microbial diversity, metabolite production, and peripheral immune regulation. These effects may reduce LPS-driven inflammatory signaling, support Treg activity, and modulate inflammatory pathways such as NF-κB, thereby limiting IL-6, TNF-α, and IL-1β signaling [83,84] [Figure 1]. Although inflammasome pathways such as NLRP3 may also contribute to inflammatory regulation, their specific role in Mediterranean diet–microbiome interactions requires evidence directly linking these dietary and microbial changes to inflammasome activity. Reduced peripheral inflammatory burden may consequently help preserve BBB function and limit downstream microglial activation and neuroinflammation [Table 2].

### 3.5. Blood–Brain Barrier Integrity

The blood–brain barrier (BBB) is a highly selective interface regulating the movement of molecules, ions, and cells between the circulation and central nervous system (CNS) [88]. Brain endothelial cells and their tight junctions form the primary barrier, supported by pericytes, astrocytes, and the basement membrane [88,89]. The BBB permits transport of essential nutrients while restricting toxins, pathogens, and inflammatory mediators, thereby maintaining CNS homeostasis [89,90].

Chronic systemic inflammation can compromise BBB integrity through exposure to pro-inflammatory cytokines, including IL-6, TNF-α, and IL-1β, and microbial products such as LPS [89]. These signals can alter endothelial tight-junction function and increase BBB permeability, facilitating inflammatory communication between the periphery and CNS [88,89]. Increased permeability may subsequently promote microglial activation, oxidative stress, and neuronal injury [80,89].

The Mediterranean diet may counter these processes through coordinated effects on the gut microbiome and systemic inflammation. Its high fiber content supports SCFA-producing bacteria, including *Faecalibacterium* and *Roseburia*, which generate acetate, propionate, and butyrate. SCFAs, particularly butyrate, can support BBB integrity and modulate inflammatory signaling [91]. Polyphenol-rich extra-virgin olive oil and omega-3 fatty acids may further reduce oxidative and inflammatory signaling, while preservation of the intestinal barrier may limit LPS translocation and peripheral immune activation [34,36,37].

BBB preservation therefore provides a downstream pathway linking Mediterranean diet–microbiome interactions to neuroprotection. BBB dysfunction may facilitate neuroinflammation and oxidative injury, impair amyloid-β clearance, and exacerbate tau pathology in AD, while also contributing to α-synuclein pathology and dopaminergic neuronal loss in PD [92,93]. Thus, maintaining BBB integrity may help limit the neuroinflammatory consequences of peripheral microbial and immune signaling [94] [Figure 1].

### 3.6. Microglial Activation

The transition from homeostatic to chronically activated microglia represents a downstream link between Mediterranean diet–microbiome interactions and neurodegeneration. Under normal conditions, microglia maintain neuronal homeostasis through immune surveillance, synaptic remodeling, and clearance of cellular debris. However, persistent inflammatory signaling can disrupt this balance, driving sustained activation and contributing to neuroinflammation, synaptic dysfunction, and neuronal injury [95].

This transition may be influenced by signals originating outside the CNS. Diet and microbiome-derived metabolites may affect microglial activation through their effects on peripheral inflammation and BBB integrity. SCFAs are particularly relevant because they can modulate inflammatory pathways and support barrier function [91,96]. By reducing peripheral inflammatory signals that promote chronic microglial activation, these effects may help preserve synaptic function and neuronal resilience [95].

Thus, microglial regulation represents a downstream convergence point for the mechanisms described above, integrating microbiome remodeling, microbial metabolites, intestinal barrier integrity, immune regulation, and BBB function. Through these interconnected pathways, Mediterranean diet adherence may reduce chronic neuroinflammation and potentially influence AD and PD risk or progression [70,97] [Table 2].

### 3.7. Oxidative Stress and Mitochondrial Function

Oxidative stress occurs when reactive oxygen species (ROS) production exceeds the capacity of antioxidant systems to neutralize them [87]. Major sources include mitochondrial electron transport and chronic inflammation, both of which can increase ROS generation [98,99,100]. Excess ROS causes lipid peroxidation, protein oxidation, and nuclear and mitochondrial DNA damage, impairing cellular function and potentially triggering apoptosis. Neurons are particularly vulnerable due to their high oxygen consumption, abundance of oxidation-prone polyunsaturated fatty acids, limited antioxidant defenses, and low regenerative capacity, making persistent oxidative stress an important contributor to neurodegeneration [98,99].

Cells counteract oxidative damage through endogenous antioxidant pathways, including nuclear factor erythroid 2–related factor 2 (Nrf2), a central regulator of redox homeostasis [101]. Under oxidative stress, Nrf2 translocates to the nucleus and activates antioxidant response element (ARE)-dependent genes encoding cytoprotective enzymes, including heme oxygenase-1 (HO-1), NAD(P)H quinone oxidoreductase 1 (NQO1), and enzymes involved in glutathione synthesis [101]. Because mitochondria are both major sources and targets of ROS, inadequate antioxidant defenses can damage mitochondrial components, impair electron transport and ATP production, and further increase ROS generation. This cycle of oxidative stress and mitochondrial dysfunction is particularly damaging to neurons and contributes to neurodegenerative processes [102].

The Mediterranean diet may counter these processes through complementary nutrient- and microbiome-mediated mechanisms. Polyphenols from extra-virgin olive oil, fruits, vegetables, and nuts can scavenge ROS and activate Nrf2-dependent antioxidant defenses, while dietary fiber supports SCFA-producing gut bacteria. In addition, unsaturated fatty acids and other bioactive compounds may reduce systemic inflammation and oxidative stress, collectively supporting redox balance and mitochondrial homeostasis [87].

These mechanisms are particularly relevant to AD and PD, where oxidative and mitochondrial dysfunction contribute to progressive neuronal injury. In AD, amyloid-β and hyperphosphorylated tau can impair mitochondrial function and increase oxidative stress, contributing to metabolic and synaptic dysfunction [103,104]. In PD, dopaminergic neurons in the substantia nigra are especially vulnerable to mitochondrial and oxidative injury, with impaired electron transport and α-synuclein accumulation contributing to neurodegeneration [103,104] [Figure 1] [Table 2]. Thus, Mediterranean diet–microbiome interactions may enhance neuronal resilience by reducing systemic inflammation, strengthening antioxidant defenses, and supporting mitochondrial function, potentially mitigating pathways involved in AD and PD progression.

## 4. Shared Mechanisms Across Alzheimer’s Disease and Parkinson’s Disease

Although AD and PD have distinct clinical and pathological features, they converge across several biological processes, including protein misfolding, mitochondrial dysfunction, oxidative stress, neuroinflammation, and impaired cellular homeostasis [105]. These shared processes overlap with pathways influenced by the Mediterranean diet and gut microbiome, including SCFA production, intestinal and blood–brain barrier integrity, microbial LPS exposure, and systemic inflammation. Thus, rather than acting on a single disease-specific pathway, Mediterranean diet-associated microbial changes may influence several interconnected mechanisms involved in both AD and PD.

### 4.1. Amyloid-β

Amyloid-β (Aβ) peptides are generated through amyloidogenic cleavage of amyloid precursor protein (APP) and can aggregate into neurotoxic oligomers, fibrils, and extracellular plaques characteristic of AD [106,107]. Aβ aggregation is influenced by several factors, including APOE, α-synuclein, and β2-microglobulin (β2M), with elevated brain B2M shown to enhance Aβ aggregation [107]. Importantly, Aβ pathology may intersect with Mediterranean diet–microbiome pathways through microbial regulation of inflammation and barrier integrity. As discussed above, Mediterranean diet adherence is associated with greater abundance of SCFA-producing bacteria and reduced pro-inflammatory microbial signaling, whereas AD-associated dysbiosis is characterized by reduced SCFA production and increased LPS-related inflammation. These microbial changes may influence systemic and neuroinflammation and blood–brain barrier integrity, providing a potential pathway linking Mediterranean diet-associated microbiome remodeling with Aβ pathology.

β2M may also interact differently with protein aggregation in PD. In contrast to its association with increased Aβ aggregation, β2M has demonstrated chaperone-like activity that maintains α-synuclein in soluble forms and inhibits its aggregation into amyloid fibrils [108] [Table 3]. These divergent effects further illustrate how common inflammatory and protein-homeostasis pathways may influence disease-specific protein aggregation.

### 4.2. Tau

Tau is a microtubule-associated protein that becomes hyperphosphorylated in AD, promoting its detachment from microtubules and aggregation into neurofibrillary tangles [109]. Aβ can further promote tau hyperphosphorylation through activation of GSK-3β and CDK-5 [110]. The potential relevance of the Mediterranean diet–microbiome axis to tau pathology again centers on microbial metabolites and inflammation. AD-associated reductions in SCFA-producing bacteria and increases in LPS-producing Gram-negative bacteria have been associated with greater inflammatory signaling and tau pathology [115]. Conversely, Mediterranean diet-associated increases in beneficial microbial metabolites and reductions in inflammatory signaling provide a plausible pathway through which dietary patterns could indirectly influence processes associated with tau pathology.

Tau may also contribute to nigrostriatal degeneration in PD independently of α-synuclein. Phosphorylated tau has been identified in individuals with mild motor deficits and PD, including in neurons exhibiting substantially reduced tyrosine hydroxylase immunoreactivity, while relatively little phosphorylated tau co-localized with α-synuclein [111] [Table 3]. Thus, inflammatory and metabolic pathways potentially modified through diet–microbiome interactions may be relevant to tau-associated pathology across both AD and PD, although direct evidence that Mediterranean diet-induced microbial changes modify tau pathology remains limited.

### 4.3. APOE ε4

APOE ε4 is a well-established genetic risk factor for late-onset AD and has been associated with pro-inflammatory processes that may contribute to neurodegeneration [112]. Growing evidence also suggests that APOE ε4 may influence susceptibility to PD, age of onset, cognitive decline, and risk of Parkinson’s disease dementia. Across individuals with AD, PD, PDD, FTD, ALS, and those without cognitive impairment, APOE ε4 carriers exhibited similar proteomic signatures characterized by activation of inflammatory immune pathways across CSF, plasma, and brain tissue [113].

This shared inflammatory phenotype is particularly relevant to the Mediterranean diet–microbiome axis because one of the proposed neuroprotective effects of Mediterranean diet-associated microbial remodeling is reduced systemic and central inflammatory signaling. APOE genotype could therefore modify the relationship between diet, microbiome-derived inflammatory signals, and neurological outcomes, although direct evidence demonstrating such an interaction remains limited. This possibility also highlights the importance of considering APOE genotype as a potential source of interindividual variability in future Mediterranean diet–microbiome studies.

### 4.4. α-Synuclein

α-synuclein aggregation is central to PD pathogenesis and contributes to dopaminergic neurodegeneration through mitochondrial dysfunction, microglial activation, and inflammatory signaling. α-synuclein aggregates induce immune responses associated with increased pro-inflammatory cytokines, including IL-1β, TNF-α, IL-6, TGF-β, and IFN-γ [71,82] [Table 3]. These inflammatory pathways provide a particularly direct connection between PD pathology and Mediterranean diet–microbiome interactions, as Mediterranean diet-associated microbial changes may reduce gut-derived inflammatory signaling and increase production of anti-inflammatory microbial metabolites.

The gut may also represent an early site of α-synuclein pathology. α-synuclein accumulation has been demonstrated within the myenteric and submucosal plexuses of the gastrointestinal tract and in colonic biopsies from prodromal PD patients, supporting the hypothesis that PD pathology may propagate through the gut–brain axis via the vagus nerve [116]. Gut dysbiosis may further promote this process: enrichment of pro-inflammatory bacteria such as Escherichia coli, reduced SCFA production, and increased inflammatory signaling may create an intestinal environment that favors α-synuclein misfolding and aggregation [116,117] [Table 3]. Mediterranean diet-associated enrichment of SCFA-producing bacteria and reductions in pro-inflammatory microbial signaling therefore provide a plausible mechanism through which diet could influence this early gut-associated pathway.

Although α-synuclein is most strongly associated with PD, it is also frequently observed as a co-pathology in AD. Approximately 59% of patients with AD in one study exhibited α-synuclein deposits, with α-synuclein-positive cases demonstrating greater cortical Aβ deposition but not an overall increase in hyperphosphorylated tau burden [114]. This overlap further supports the possibility that interventions targeting shared upstream processes, including microbiome-associated inflammation, may have relevance across both diseases.

### 4.5. Dysbiosis

Gut dysbiosis therefore represents a potential point of convergence between Mediterranean diet exposure and the shared neurodegenerative mechanisms described above. In PD, enrichment of pro-inflammatory organisms such as Escherichia coli may promote intestinal inflammation and α-synuclein misfolding, while reduced SCFA production may further contribute to chronic inflammatory signaling [116,117].

Similarly, patients with AD have exhibited reductions in beneficial SCFA-producing bacteria, including *Faecalibacterium prausnitzii*, *Roseburia hominis*, *Eubacterium rectale*, and *Ruminococcus*, alongside increases in pro-inflammatory bacteria such as Escherichia coli and Klebsiella. Reduced production of butyrate, acetate, and propionate may compromise intestinal and blood–brain barrier integrity, while increased Gram-negative bacterial abundance can increase LPS-related inflammatory signaling. These alterations have been associated with increased amyloid plaque burden, tau pathology, and cognitive decline. Dysbiosis may also disrupt bile acid metabolism, with altered bile acid profiles correlating with poorer memory and executive function and pathways involved in neuroinflammation and mitochondrial dysfunction [115].

These findings connect the disease-specific mechanisms described above with the microbial effects associated with Mediterranean diet adherence. By promoting SCFA-producing microbial communities and microbial metabolites while potentially reducing LPS-related and other pro-inflammatory signaling, the Mediterranean diet may influence upstream processes linked to Aβ, tau, and α-synuclein pathology. However, most human evidence currently supports associations among diet, microbiome composition, and neurological outcomes rather than demonstrating that Mediterranean diet-induced microbial remodeling directly modifies these proteinopathies.

## 5. Human Evidence and Translational Relevance

Although mechanistic and experimental studies have identified pathways through which the Mediterranean diet may promote neuroprotection, human studies are essential to determining whether these mechanisms translate into clinically meaningful outcomes. Observational studies, longitudinal cohorts, and randomized controlled trials (RCTs) provide complementary evidence by examining dietary patterns across populations, over time, and under controlled intervention conditions. Together, these approaches help establish the association, temporal relationship, and potential causal mechanisms linking Mediterranean diet adherence with gut microbiome composition, inflammation, cognitive aging, and neurodegenerative disease risk.

### 5.1. Observational Studies

Observational studies provide population-level evidence by examining associations between habitual dietary patterns and health outcomes under real-world conditions. Their large and diverse populations allow researchers to evaluate relationships between long-term Mediterranean diet adherence and chronic conditions, including cognitive decline and neurodegenerative disorders [118,119]. However, because dietary exposures are not experimentally controlled, these studies cannot establish causality and remain vulnerable to residual confounding, reverse causality, and dietary recall bias. Despite these limitations, observational findings provide an important foundation for identifying associations that can be evaluated more rigorously through longitudinal and interventional studies.

### 5.2. Longitudinal Cohorts

Longitudinal cohort studies extend observational evidence by tracking dietary, lifestyle, and health characteristics over extended periods, often beginning years before the onset of neurodegenerative disease. The Rush Memory and Aging Project illustrates the value of prospective cohort designs that combine repeated cognitive assessment with detailed clinical and neuropathological characterization, enabling investigators to evaluate risk factors and trajectories that precede dementia [120]. More broadly, longitudinal dietary cohorts have been essential for examining whether sustained Mediterranean-style dietary patterns precede differences in cognitive decline rather than merely reflecting health changes after disease onset. Nevertheless, these studies remain observational and therefore cannot fully eliminate residual confounding or establish that dietary exposure itself causes the observed neurological outcomes.

### 5.3. Randomized Controlled Trials

RCTs provide stronger evidence for causal effects by prospectively assigning participants to dietary interventions. The PREDIMED trial demonstrated that a Mediterranean diet supplemented with extra-virgin olive oil or nuts reduced debilitating major health events, providing strong evidence for the cardiometabolic benefits of this dietary pattern [121]. More recent intervention studies have begun examining whether Mediterranean diet adherence can also modify gut microbial composition and metabolic activity [23,122,123]. In particular, dietary fiber and other fermentable substrates can promote the production of SCFAs, including acetate, propionate, and butyrate, which can support intestinal barrier integrity and regulate immune and inflammatory signaling [50]. These microbiome-derived metabolites therefore represent a potential biological link between Mediterranean diet adherence, reduced systemic inflammation, and downstream effects on the microbiota–gut–brain axis. However, evidence remains limited regarding whether these microbiome-mediated changes translate into sustained reductions in Alzheimer’s disease, Parkinson’s disease, or other neurodegenerative outcomes. Future RCTs should therefore integrate dietary interventions with longitudinal microbiome profiling, inflammatory biomarkers, neuroimaging, and cognitive or neurological assessments to determine whether microbiome-mediated pathways contribute to clinically meaningful neuroprotection.

## 6. Critical Appraisal of Current Evidence

### 6.1. Heterogeneity of Mediterranean Diet Assessments

Although the Mediterranean diet is generally characterized by a high intake of fruits, vegetables, legumes, whole grains, nuts, fish, and olive oil, there is no single standardized definition or scoring system used across studies. Commonly used measures include the Mediterranean Diet Score, the alternate Mediterranean Diet Score, the Mediterranean Diet Adherence Screener, and other population-specific adaptations. These tools differ in the dietary components included, the weighting assigned to each component, the cutoff values used to define adherence, and the treatment of foods such as dairy products, alcohol, potatoes, refined grains, and olive oil. Some scores evaluate intake relative to population-specific medians, whereas others use fixed recommended servings, meaning that an individual classified as highly adherent under one scoring system may not receive the same classification under another. Reviews of Mediterranean diet scoring systems have emphasized that this flexibility has contributed to inconsistencies in how adherence is defined and limits direct comparison across Mediterranean and non-Mediterranean populations [124].

This heterogeneity is especially relevant when evaluating microbiome-mediated mechanisms because different versions of the Mediterranean diet may provide substantially different levels of fiber, polyphenols, unsaturated fatty acids, and other microbial substrates. For example, a score that heavily weights olive oil and fish intake may represent a biologically different dietary exposure from one primarily driven by fruit, vegetable, and legume consumption, despite both being classified as high Mediterranean diet adherence. These differences may result in distinct microbial and metabolic responses and could partly explain inconsistencies in the bacterial taxa, short-chain fatty acid concentrations, and inflammatory outcomes reported across studies. Therefore, future studies should clearly report the scoring system used, the individual dietary components contributing to the score, and the absolute intake of major mechanistically relevant nutrients. Greater standardization of Mediterranean diet definitions would improve comparisons across studies and help determine which specific dietary components or combinations are most strongly associated with microbiome remodeling and neuroprotection in AD and PD.

### 6.2. Heterogeneity in Microbiome Sequencing and Analysis

Considerable methodological variation also exists in how the gut microbiome is characterized. Many studies use 16S ribosomal RNA gene sequencing because it is relatively accessible and cost-effective; however, this approach generally provides limited species- or strain-level resolution and does not directly measure the functional genes or metabolic activity of the microbial community. In comparison, shotgun metagenomic sequencing can provide greater taxonomic resolution and characterize the functional potential of the microbiome, but it is more expensive and remains less commonly used. Consequently, studies using different sequencing approaches may not be measuring equivalent features of the microbial ecosystem [125].

The obtained results may also be influenced by the region of the 16S gene selected for amplification, sequencing depth, DNA extraction procedures, reference databases, normalization strategies, diversity measures, and bioinformatic pipelines. This variation can produce different estimates of microbial abundance even when similar biological samples are analyzed. The problem was illustrated by a recent scoping review of 1262 diet–microbiome studies, which identified extensive heterogeneity in dietary assessment and microbiome methods across the field. A study of different methods used to connect diet and the gut microbiome found a wide variety, with over 100 different dietary collection methods across almost 300 studies [126].

### 6.3. Stool Collection, Storage, and Temporal Variability

Differences in stool collection and sample processing represent another important source of variability. Studies differ in whether samples are collected at home or in a clinical setting, the time between defecation and freezing, the temperature at which samples are stored, the use of stabilization solutions, transportation conditions, and the DNA extraction kits used. Each of these factors may influence the relative abundance and detectability of microbial organisms, potentially creating apparent biological differences that instead reflect preanalytical variation. Methodological reviews therefore recommend standardized collection, preservation, storage, processing, and reporting procedures in diet–microbiome studies [127].

Another limitation is that many studies rely on a single stool sample to characterize an individual’s microbiome. However, microbial composition and function can vary over time in response to short-term dietary intake, medication exposure, illness, bowel habits, travel, and other environmental factors. A single sample may therefore fail to represent an individual’s usual microbial state or capture the dynamic response to a Mediterranean dietary intervention. The timing of stool collection relative to dietary assessment is also important. Microbiome profiles collected weeks or months apart from food-frequency questionnaires may not accurately reflect the microbial community associated with the reported dietary exposure. The large methodological scoping review of diet–microbiome studies specifically identified substantial variation in the timing and frequency of both dietary and biological sampling [126].

These concerns are particularly relevant to AD and PD because disease progression itself may alter bowel function, diet, medication use, and microbial composition. Repeated stool collection before, during, and after dietary interventions would provide a more reliable assessment of microbial stability and change over time. Ideally, stool samples should be obtained alongside repeated dietary measurements, circulating and fecal metabolite analyses, inflammatory markers, and neurological outcomes. Such designs would help distinguish sustained microbiome remodeling from short-term fluctuations and clarify whether microbial changes precede or follow changes in disease-related biomarkers.

Reverse causality is an additional concern, particularly in studies of individuals with established AD or PD. Neurodegenerative disease itself may alter dietary intake, appetite, food preferences, physical activity, medication use, and gastrointestinal function, each of which can independently influence gut microbial composition. Gastrointestinal dysfunction is particularly relevant in PD, where constipation and altered intestinal motility may occur early in the disease course. Consequently, microbiome differences observed in individuals with neurodegenerative disease may represent a consequence of disease-related physiological and behavioral changes rather than an upstream contributor to disease pathogenesis. Prospective studies beginning before clinical disease onset are therefore needed to determine the temporal sequence among dietary exposure, microbiome alterations, and neurodegenerative changes.

### 6.4. Why Causality Remains Difficult to Establish

Taken together, these methodological limitations explain why a causal Mediterranean diet–microbiome–brain pathway has not yet been firmly established in humans. Current research has independently demonstrated associations between Mediterranean diet adherence and microbial composition, between Mediterranean diet adherence and cognitive or neurological outcomes, and between dysbiosis, inflammation, and neurodegenerative disease. However, relatively few studies have examined the following entire proposed sequence within the same participants:

***Mediterranean diet adherence → sustained microbiome remodeling → altered microbial metabolite production → improved intestinal and blood–brain barrier integrity → reduced systemic and central inflammation → modification of Aβ, tau, or α-synuclein pathology → improved clinical outcomes.***

Each arrow within this pathway is supported by varying levels of observational, interventional, or preclinical evidence, but connecting all of these stages in a single causal mechanism remains challenging. Reviews of diet–microbiome research have emphasized that methodological heterogeneity, inconsistent dietary measurement, limited longitudinal sampling, inadequate control of confounders, and the frequent reliance on taxonomic rather than functional microbiome data continue to limit reproducibility and causal inference.

Importantly, these associations should not be interpreted as evidence that Mediterranean diet adherence or microbiome alterations directly cause improved neurological outcomes. Mediterranean diet adherence often occurs alongside other health-promoting behaviors, including greater physical activity, lower rates of smoking, and broader differences in overall lifestyle. Socioeconomic factors, including education, income, healthcare access, and food availability, may also influence both dietary adherence and neurological outcomes. Although observational studies attempt to adjust for these variables, residual confounding remains difficult to eliminate and may partially account for associations between Mediterranean diet adherence, microbiome composition, and neurodegenerative risk. Together with the potential for reverse causality described above, these sources of residual confounding make it difficult to determine whether observed microbiome differences contribute to neurodegeneration or instead reflect broader lifestyle factors and disease-related changes.

Randomized dietary trials strengthen causal inference regarding the effects of the Mediterranean diet, but even these studies may not establish that the microbiome mediates neurological benefits. To demonstrate mediation, a study would need to show that the intervention changes microbial composition or function, that these microbial changes occur before and predict changes in neurological outcomes, and that the estimated dietary benefit is at least partly explained by the microbial response. Future studies should therefore combine randomized or carefully designed longitudinal interventions with repeated dietary assessments, stool sampling, metagenomic and metabolomic analyses, measures of intestinal and BBB integrity, inflammatory profiling, and disease-specific biomarkers. Formal mediation analyses and, where feasible, microbiome-targeted experimental approaches could then evaluate whether microbial changes are necessary contributors to the effects of the Mediterranean diet.

## 7. Knowledge Gaps and Future Directions

### 7.1. Limited Evidence for Microbiome-Mediated Causality

Most human studies show that Mediterranean diet adherence is associated with favorable microbial profiles and better neurological outcomes, but they do not establish that microbiome remodeling is responsible for the benefit. For example, a recent prospective study linked Mediterranean diet adherence and a 20-taxon microbiome signature with slower cognitive decline over six years. However, the microbiome was primarily profiled at baseline, the study population consisted of adults with overweight or obesity and metabolic syndrome rather than biomarker-confirmed AD, and experimental validation was still needed [128].

Although Mediterranean diet adherence has been associated with favorable changes in gut microbial composition, reduced systemic inflammation, and improved cognitive or neurological outcomes, the extent to which these benefits are actually mediated by the gut microbiome remains unclear. Much of the current human evidence is observational and demonstrates that Mediterranean diet adherence, microbial composition, and brain health are related but does not establish the direction of these relationships or determine whether microbiome remodeling is necessary for neuroprotection. Individuals who adhere more closely to a Mediterranean diet may also be more physically active, have better cardiometabolic health, use fewer tobacco products, have greater access to healthcare, or engage in other behaviors that independently reduce the risk of AD and PD. Similarly, a healthier microbiome may reflect broader differences in lifestyle, medication use, geography, or baseline health rather than a direct biological response to the diet itself. Reverse causality is also possible, particularly in established neurodegenerative disease, because cognitive impairment, gastrointestinal dysfunction, reduced mobility, altered appetite, and medication use may change both dietary intake and microbial composition.

A major unanswered question is therefore whether the Mediterranean diet protects the brain specifically by altering microbial ecology and function or whether dietary adherence, microbiome profiles, and neurological health simply co-vary with a broader pattern of healthy aging. Addressing this question requires studies designed to test mediation rather than association alone. Future longitudinal and interventional studies should collect repeated dietary and microbiome measurements before and after Mediterranean diet exposure and simultaneously assess microbial metabolites, intestinal permeability, systemic inflammation, blood–brain barrier function, disease-specific biomarkers, and cognitive or motor outcomes. Randomized dietary trials could further stratify participants according to baseline microbiome characteristics, such as microbial diversity, abundance of SCFA-producing taxa, or fecal SCFA concentrations, to determine whether these features predict or modify the response to Mediterranean diet interventions. Formal mediation analyses could then determine whether changes in microbial taxa or functions—such as increased SCFA production, altered bile acid metabolism, reduced LPS-related signaling, or enhanced polyphenol metabolism—account for part of the relationship between the diet and neurological outcomes. Ideally, these studies would also evaluate whether the neuroprotective effect of the diet is weakened or absent in individuals who do not exhibit the expected microbial response. Such designs would help identify microbiome-defined responder subgroups while providing stronger evidence for whether microbial changes precede and mediate neurological benefits. By integrating the currently fragmented evidence across diet, microbiome function, immune regulation, barrier integrity, and neurodegenerative pathology, this review clarifies which mechanisms are directly supported, which remain inferential, and where future research is needed to establish a causal Mediterranean diet–microbiome–brain pathway in AD and PD.

### 7.2. Greater Mechanistic Uncertainty in Parkinson’s Disease than Alzheimer’s Disease

Compared with AD, there is substantially less direct evidence regarding the mechanistic effects of adhering to the Mediterranean diet on PD. Most studies evaluating the Mediterranean diet in PD have focused on disease incidence, prodromal symptoms, or overall disease risk rather than determining how the diet influences the biological pathways underlying PD progression. A recent systematic review and meta-analysis including 12 observational studies found that greater adherence to the Mediterranean diet was associated with a 25% lower risk of PD and a 33% lower risk of prodromal PD, suggesting that dietary modification may play an important role in disease prevention. However, these findings were derived from observational cohort, case–control, and cross-sectional studies and therefore cannot establish causality or determine the biological mechanisms responsible for the observed associations. Although anti-inflammatory effects, improved mitochondrial function, and modulation of the gut microbiome have all been proposed as potential mechanisms through which the Mediterranean diet may reduce PD risk, these pathways remain largely hypothetical in humans and have not been directly demonstrated in longitudinal mechanistic studies [129,130].

This represents an important gap in the current literature. Unlike AD, for which an increasing number of studies have begun integrating dietary interventions with biomarkers of amyloid-β, tau, neuroinflammation, and cognitive decline, comparable mechanistic studies in PD remain limited. Few prospective studies have simultaneously evaluated Mediterranean diet adherence, longitudinal alterations in the gut microbiome and microbial metabolites, systemic inflammatory responses, and disease-specific measures of PD pathology. Consequently, it remains unclear whether Mediterranean diet-induced microbial remodeling directly alters α-synuclein aggregation, dopaminergic neurodegeneration, or disease progression, or whether the observed reductions in PD risk reflect broader improvements in metabolic health rather than disease-specific neuroprotective mechanisms.

Future research should therefore move beyond epidemiological associations toward longitudinal mechanistic studies that integrate dietary assessment with repeated stool sampling, shotgun metagenomics, microbial metabolomics, inflammatory profiling, intestinal and blood–brain barrier biomarkers, and validated measures of PD pathology. Incorporating biomarkers such as α-synuclein seed amplification assays, enteric or peripheral α-synuclein measurements, dopaminergic imaging, and longitudinal assessments of both motor and non-motor symptoms would help determine whether Mediterranean diet-induced microbiome remodeling directly modifies PD pathogenesis. Such studies will be essential for distinguishing association from causation and identifying the biological pathways through which dietary interventions may influence PD development and progression.

### 7.3. Probiotics, Prebiotics, and Microbiome-Based Therapeutics in Conjunction with the MD

Growing evidence suggests that probiotics, prebiotics, synbiotics, and other microbiome-targeted interventions may represent promising strategies for reducing neuroinflammation and supporting cognitive and neurological health. Several clinical studies have reported improvements in cognitive performance among individuals with mild cognitive impairment (MCI) following supplementation with probiotic strains including Lactobacillus rhamnosus, *Bifidobacterium* breve MCC1274, and *Bifidobacterium* species, while animal studies have demonstrated reductions in amyloid-β deposition, neuroinflammation, oxidative stress, and improvements in memory and synaptic plasticity [131,132,133,134,135,136]. Similarly, probiotic supplementation in PD has been associated with improvements in constipation, motor and non-motor symptoms, and reductions in pro-inflammatory cytokines including IL-1, IL-6, TNF-α, and IFN-γ, while preclinical studies suggest that specific strains such as Lactobacillus plantarum PS128 may preserve dopaminergic neurons, increase dopamine concentrations, and reduce microglial activation [137,138,139].

Despite these encouraging findings, substantial uncertainties remain regarding the clinical translation of microbiome-based interventions. Existing studies differ considerably in probiotic species, strain selection, dosage, formulation, treatment duration, participant characteristics, and clinical endpoints, making direct comparisons difficult and limiting the ability to identify the most effective therapeutic strategies. Importantly, probiotic effects are highly strain-specific, and beneficial effects observed with one organism cannot be generalized to other members of the same bacterial genus or species. Furthermore, many clinical studies have been conducted in relatively small cohorts with short follow-up periods, making it difficult to determine whether improvements in cognition, inflammatory biomarkers, or gastrointestinal symptoms translate into sustained modification of disease progression.

Another major limitation is that probiotic studies frequently evaluate clinical outcomes without determining whether the administered organisms successfully colonize the gut or produce the anticipated functional changes in microbial metabolism. Improvements in microbial diversity or bacterial abundance do not necessarily correspond to increased production of neuroprotective metabolites such as SCFAs, indole derivatives, or beneficial bile acids. Likewise, few studies simultaneously measure microbial composition, microbial metabolites, intestinal permeability, systemic inflammation, blood–brain barrier integrity, and disease-specific biomarkers, making it difficult to establish the mechanistic pathways through which probiotics exert their effects. Future clinical trials should therefore integrate shotgun metagenomics, metabolomics, inflammatory profiling, and validated biomarkers of AD and PD pathology to determine whether microbiome-targeted therapies directly modify the biological mechanisms underlying neurodegeneration.

An additional challenge is the marked interindividual variability in probiotic responsiveness. Host genetics, baseline microbiome composition, dietary habits, medication use, age, and metabolic status all influence whether administered microorganisms successfully engraft and produce meaningful metabolic changes. Consequently, identical probiotic formulations may produce substantially different biological responses across individuals. This variability suggests that future therapeutic approaches may need to move beyond standardized probiotic supplementation toward precision microbiome-based interventions tailored to an individual’s baseline microbial ecology and metabolic profile.

Finally, probiotics should not be viewed as isolated interventions but rather as one component of a broader dietary strategy. The Mediterranean diet provides dietary fiber, polyphenols, omega-3 fatty acids, and other substrates that support the growth and metabolic activity of beneficial microorganisms. Without these substrates, probiotic organisms may have limited ability to colonize or produce sustained functional changes. Consequently, future studies should investigate the combined effects of Mediterranean dietary adherence with probiotics, prebiotics, synbiotics, and postbiotics to determine whether these interventions act synergistically to improve microbial function, reduce neuroinflammation, preserve blood–brain barrier integrity, and ultimately delay the onset or progression of AD and PD. Such studies may provide the foundation for precision nutrition strategies that combine dietary modification with targeted microbiome-based therapeutics to prevent or slow neurodegenerative disease.

### 7.4. Artificial Intelligence, Digital Health, and Precision Nutrition

Recent advances in artificial intelligence (AI), machine learning, and digital health technologies provide new opportunities to better understand the complex relationship between diet, the gut microbiome, and neurodegeneration. Traditional nutritional studies have largely relied on food frequency questionnaires, dietary recalls, or food diaries, which are subject to recall bias and often fail to capture day-to-day dietary variability. Likewise, microbiome studies frequently rely on single stool samples that may not adequately reflect dynamic changes in microbial composition and function over time. These methodological limitations make it difficult to accurately characterize long-term dietary exposures and determine how changes in diet influence microbiome-mediated mechanisms underlying AD and PD [140].

AI-based dietary assessment tools have the potential to overcome many of these limitations by providing continuous, objective, and personalized monitoring of dietary intake. Computer vision algorithms capable of identifying foods from smartphone photographs, estimating portion size, and calculating nutrient intake have advanced considerably over the past decade, while wearable sensors capable of detecting eating behaviors through jaw motion, swallowing, hand-to-mouth movements, or other physiological signals are increasingly being incorporated into dietary monitoring platforms. These technologies substantially reduce participant burden while enabling real-time, high-resolution dietary assessment that is difficult to achieve using conventional self-report methods [141].

The integration of AI with wearable health technologies may further expand opportunities for precision nutrition. Continuous monitoring of physical activity, sleep quality, heart rate variability, continuous glucose measurements, and other physiological parameters can be integrated with dietary intake, gut microbiome sequencing, metabolomics, and clinical biomarkers to generate individualized models of diet–microbiome interactions. Machine learning algorithms are increasingly being developed to combine these multimodal datasets and predict personalized dietary responses based on microbial composition, metabolic status, and host physiology, representing an emerging framework for precision nutrition [142].

Beyond improving dietary assessment, AI may facilitate individualized dietary recommendations rather than generalized nutritional guidelines. Future algorithms may predict which individuals are most likely to increase short-chain fatty acid production following increased dietary fiber intake, identify patients who may benefit from specific probiotic or prebiotic interventions, or determine how host factors such as APOE genotype, baseline microbiome composition, and metabolic health influence responsiveness to Mediterranean diet interventions. Such approaches could enable precision nutrition strategies that optimize microbiome-mediated neuroprotection and support AD and PD prevention [143].

Although these technologies are promising, several important challenges remain. Existing AI models require large, diverse, longitudinal datasets for robust training and external validation, and many current algorithms have been developed using relatively homogeneous study populations. Standardization of dietary assessment methods, microbiome sequencing, wearable-derived physiological measures, and clinical outcomes will also be necessary before these approaches can be translated into routine clinical practice. Furthermore, issues related to algorithm interpretability, privacy, and equitable implementation across diverse populations must be addressed before AI-driven precision nutrition can become a clinically applicable strategy for preventing neurodegenerative disease [140].

## 8. Conclusions

The Mediterranean diet represents one of the most promising lifestyle strategies for promoting healthy cognitive aging and reducing the risk of neurodegenerative disease. Growing evidence suggests that its neuroprotective effects extend beyond the direct actions of individual nutrients and may be mediated, at least in part, through remodeling of the gut microbiome. By promoting beneficial microbial communities and increasing the production of neuroprotective metabolites such as short-chain fatty acids, the Mediterranean diet may preserve intestinal and blood–brain barrier integrity, reduce systemic inflammation and neuroinflammation, improve immune regulation, and attenuate pathological processes involved in both Alzheimer’s disease and Parkinson’s disease. Importantly, this review highlights that many of these mechanisms, including dysbiosis, barrier dysfunction, chronic inflammation, oxidative stress, mitochondrial dysfunction, and pathological protein aggregation, are shared across AD and PD, suggesting that modulation of the gut microbiome may represent a promising target for future preventive or therapeutic investigation across neurodegenerative disorders.

Despite these advances, important knowledge gaps remain. Although epidemiological, clinical, and preclinical studies collectively support a biologically plausible relationship between Mediterranean diet adherence, gut microbial function, and neuroprotection, the causal pathways linking these processes have not yet been fully established. Future longitudinal and interventional studies integrating dietary interventions with repeated microbiome profiling, metabolomics, disease-specific biomarkers, and advanced computational approaches will be essential for determining whether microbiome remodeling directly mediates the neurological benefits of the Mediterranean diet. As precision nutrition, microbiome science, artificial intelligence, and biomarker discovery continue to advance, these complementary fields have the potential to transform personalized strategies for preventing or delaying AD and PD. By integrating nutrition with microbial and molecular mechanisms, the Mediterranean diet may ultimately provide an accessible, safe, and scalable approach for promoting brain health across the lifespan.

## Figures and Tables

**Figure 1:**
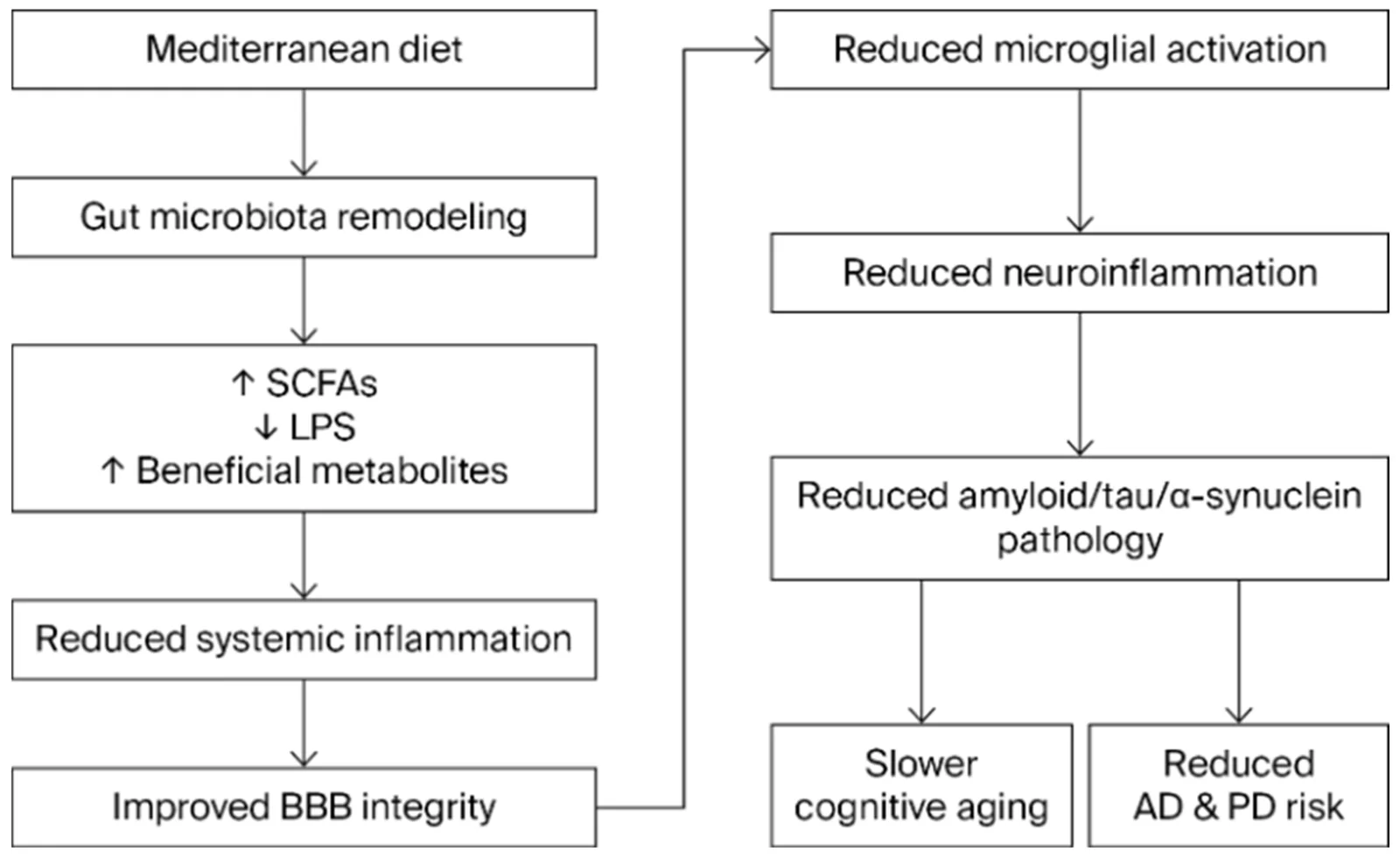
Conceptual model illustrating the proposed microbiome-mediated mechanisms by which the Mediterranean diet promotes neuroprotection and may reduce the risk of neurodegenerative disease. Adherence to the Mediterranean diet promotes gut microbiota remodeling by enriching beneficial microbial taxa and increasing the production of neuroprotective metabolites, including short-chain fatty acids (SCFAs), while reducing lipopolysaccharides (LPSs) and other pro-inflammatory microbial products. These changes improve intestinal barrier integrity, reduce systemic inflammation, preserve blood–brain barrier (BBB) function, and attenuate microglial activation and neuroinflammation. Collectively, these interconnected mechanisms may limit the accumulation of Alzheimer’s disease- and Parkinson’s disease-related neuropathology, including amyloid-β, hyperphosphorylated tau, and α-synuclein, thereby supporting healthy cognitive aging and reducing the risk of Alzheimer’s disease and Parkinson’s disease.

**Table 1:** Key: List of abbreviations found within the text.

Full Word	Abbreviation	Primary Function/Relevance
Alzheimer’s Disease	AD	Progressive neurodegenerative disease characterized by cognitive decline and neuropathological features including amyloid-β plaques, tau pathology, synaptic dysfunction, and neuronal loss.
Parkinsons disease	PD	Progressive neurodegenerative disease primarily involving degeneration of dopaminergic neurons and α-synuclein pathology, resulting in motor and non-motor symptoms.
Short Chain Fatty Acids	SCFA(s)	Microbial metabolites, primarily acetate, propionate, and butyrate, produced through fermentation of dietary fiber; support intestinal barrier integrity and influence immune, metabolic, and gut–brain signaling.
Transforming growth factor-β	TGF-β	Regulatory cytokine involved in immune regulation, tissue repair, cellular differentiation, and control of inflammatory responses.
Interleukin-10	IL-10	Anti-inflammatory cytokine that suppresses excessive immune activation and limits production of several pro-inflammatory mediators.
Gamma-aminobutyric acid	GABA	Major inhibitory neurotransmitter in the central nervous system that regulates neuronal excitability and contributes to neural signaling and network stability.
C-reactive protein	CRP	Acute-phase protein produced primarily by the liver in response to inflammatory signaling; commonly used as a circulating marker of systemic inflammation.
Lipopolysaccharides	LPS	Component of the outer membrane of Gram-negative bacteria that can activate innate immune signaling and promote inflammatory responses, particularly when intestinal barrier integrity is impaired.
Toll-like receptor 4	TLR4	Innate immune pattern-recognition receptor that recognizes LPS and activates downstream inflammatory signaling pathways, including NF-κB.
Interleukin-6	IL-6	Pleiotropic cytokine involved in immune regulation and acute-phase responses; persistent elevations are commonly associated with chronic systemic inflammation.
Tumor necrosis factor-α	TNF-α	Pro-inflammatory cytokine that regulates immune activation and can contribute to chronic inflammation, endothelial dysfunction, and neuroinflammatory signaling when persistently elevated.
Interleukin-1β	IL-1β	Potent pro-inflammatory cytokine involved in innate immune activation, fever, inflammatory signaling, and neuroimmune responses.
Blood–brain barrier	BBB	Selective interface between the circulation and central nervous system that regulates molecular and cellular entry into the brain and helps maintain neural homeostasis.
Nuclear Factor Kappa B	NF-κB	Transcription factor that regulates expression of numerous genes involved in inflammation, immune responses, cellular stress, and survival.
Central Nervous System	CNS	The brain and spinal cord; integrates sensory information and coordinates neurological, cognitive, behavioral, and motor functions.

**Table 2:** Major components of the Mediterranean diet, their effects on the gut microbiome, proposed microbiome-mediated neuroprotective mechanisms, associated cognitive outcomes, and supporting evidence. Evidence linking major components of the Mediterranean dietary pattern with alterations in gut microbial composition and function and their proposed downstream effects on intestinal barrier integrity, systemic inflammation, blood–brain barrier function, neuroinflammation, and cognitive and neurodegenerative outcomes. Proposed mechanisms represent pathways supported to varying degrees by human observational, interventional, mechanistic, and preclinical evidence and should not necessarily be interpreted as established causal relationships.

Mediterranean Diet Component	Primary Gut Microbiome Effects	Proposed Neuroprotective Mechanisms	Cognitive/Neurodegenerative Outcomes	Type of Evidence and Citation
Dietary fiber (whole grains, fruits, vegetables, legumes)	↑ SCFA-producing bacteria (*Faecalibacterium prausnitzii*, *Roseburia*, *Bifidobacterium*); ↑ microbial diversity	↑ Butyrate, acetate, and propionate production; improved intestinal and BBB integrity; reduced systemic inflammation; immune regulation	Slower cognitive decline; reduced AD and PD risk	Human observational studies, RCTs, animal studies [28,29]
Polyphenols (berries, tea, cocoa, fruits, vegetables)	↑ Lactobacillus, *Bifidobacterium*; microbial production of phenolic metabolites	Antioxidant activity; reduced oxidative stress; enhanced mitochondrial function; suppression of neuroinflammation	Improved cognition; reduced neuroinflammation; potential protection against AD and PD	Human observational studies, intervention studies, experimental studies [33]
Extra-virgin olive oil	↑ Beneficial bacteria; ↑ SCFA-producing taxa	Reduced oxidative stress; improved gut barrier integrity; decreased inflammatory cytokines; improved endothelial function	Improved cognitive performance; reduced dementia risk	RCTs (PREDIMED), cohort studies, mechanistic studies [8,34]
Omega-3 fatty acids (fish)	↑ *Bifidobacterium*, Lactobacillus; ↓ pro-inflammatory taxa	Production of specialized pro-resolving mediators (SPMs); reduced neuroinflammation; improved membrane integrity and neuronal signaling	Lower cognitive decline; reduced neurodegenerative risk	Human intervention studies, observational studies, animal models [39,40]
Whole grains	↑ SCFA production; ↑ microbial diversity	Enhanced gut barrier integrity; improved glucose metabolism; immune homeostasis	Reduced inflammation associated with cognitive aging	Human observational and mechanistic studies [45]
Nuts	↑ Faecalibacterium, *Bifidobacterium*, Lactobacillus	Increased SCFA production; antioxidant and anti-inflammatory effects; improved metabolic function	Better cognitive performance; reduced dementia risk	RCTs, cohort studies [46,47]
Legumes	↑ Fiber fermentation; ↑ SCFA-producing bacteria	Improved intestinal barrier function; reduced endotoxemia; immune regulation	Reduced chronic inflammation associated with cognitive aging	Human observational and mechanistic studies [49,50]
Fermented dairy (yogurt, kefir)	Introduction of beneficial microbes; improved microbial diversity	Modulation of immune responses; production of bioactive peptides; improved intestinal homeostasis	Potential cognitive benefits through gut–brain axis modulation	Human observational studies; limited intervention studies [51,52,53,55]
Overall Mediterranean dietary pattern	↑ Microbial diversity; ↑ SCFAs and beneficial metabolites; ↓ dysbiosis and LPS-producing bacteria	Reduced gut permeability, systemic inflammation, BBB dysfunction, microglial activation, oxidative stress, and pathological protein aggregation	Slower cognitive aging; lower incidence of Alzheimer’s disease, Parkinson’s disease, and dementia	Randomized controlled trials, longitudinal cohorts, observational studies, mechanistic and animal studies

Up arrows symbolize increase, down arrows symbolize decrease.

**Table 3:** Shared mechanisms across AD and PD. The table summarizes overlapping molecular, inflammatory, immune, and gut–brain mechanisms implicated in the pathogenesis and progression of Alzheimer’s disease (AD) and Parkinson’s disease (PD), along with the supporting references and corresponding evidence types.

Shared Mechanism	Alzheimer’s Disease	Parkinson’s Disease	Type of Evidence and Citation
β2-Microglobulin (B2M)	Elevated B2M promotes Aβ aggregation; lowering peripheral B2M improves cognition in mouse models.	B2M exhibits chaperone-like activity that inhibits α-synuclein fibrillization and promotes soluble, non-toxic species.	Preclinical [107,108]
Tau pathology	Hyperphosphorylated tau forms neurofibrillary tangles following Aβ-induced kinase activation, contributing to neurodegeneration.	Phosphorylated tau is present in early PD, including individuals without Lewy pathology, and is associated with dopaminergic degeneration and reduced TH expression.	Human observational + mechanistic/preclinical [109,110,111]
APOE ε4-mediated immune dysregulation	APOE ε4 is the strongest genetic risk factor for late-onset AD and promotes chronic neuroinflammation.	APOE ε4 is associated with increased PD susceptibility, earlier onset, cognitive decline, and PDD, while carriers share a conserved inflammatory proteomic signature.	Human observational [112,113]
α-Synuclein/Aβ overlap	Approximately 59% of AD brains exhibit α-synuclein pathology, which is associated with greater cortical Aβ deposition.	α-Synuclein aggregation is central to PD pathogenesis and promotes mitochondrial and lysosomal dysfunction, as well as inflammation.	Human observational + mechanistic [71,82,114]
Neuroinflammation	Aβ, tau, APOE ε4, and gut-derived inflammatory mediators promote chronic neuroinflammation and neuronal injury.	α-Synuclein activates microglia and inflammatory pathways, increasing IL-1β, IL-6, TNF-α, IFN-γ, and other cytokines. APOE ε4 similarly promotes a conserved inflammatory immune signature.	Human observational, systematic review, and mechanistic studies [71,82,113,115]
Gut dysbiosis/gut–brain axis	Loss of SCFA-producing bacteria and enrichment of pro-inflammatory bacteria increase LPS production, BBB dysfunction, neuroinflammation, Aβ deposition, tau pathology, and cognitive decline.	Dysbiosis similarly reduces SCFAs, increases inflammatory bacteria such as E. coli, promotes α-synuclein misfolding and aggregation, and supports propagation through the gut–brain axis.	Systematic review, observational, and mechanistic studies [115,116,117]
Chronic immune activation	Peripheral and central immune activation contribute to disease progression through cytokine production and BBB dysfunction.	Similar innate and adaptive immune activation occurs in PD, including activation of monocytes, NK cells, γδ T cells, and memory CD8 T cells.	Human observational + mechanistic [71,113]

## Data Availability

No new data were created or analyzed in this study. Data sharing is not applicable to this article.
